# Transcriptomic Analysis Reveals Genes Mediating Salt Tolerance through Calcineurin/CchA-Independent Signaling in* Aspergillus nidulans*

**DOI:** 10.1155/2017/4378627

**Published:** 2017-08-20

**Authors:** Sha Wang, Hongchang Zhou, Jun Wu, Jiangyu Han, Shasha Li, Shengwen Shao

**Affiliations:** ^1^Department of Pathogen and Immunity, School of Medicine, Huzhou University, Zhejiang, China; ^2^Traditional Chinese Medicine Hospital of Huangyan, Zhejiang, China

## Abstract

Adaptation to changes in the environment is crucial for the viability of all organisms. Although the importance of calcineurin in the stress response has been highlighted in filamentous fungi, little is known about the involvement of ion-responsive genes and pathways in conferring salt tolerance without calcium signaling. In this study, high-throughput RNA-seq was used to investigate salt stress-induced genes in the parent, Δ*cnaB*, and Δ*cnaB*Δ*cchA* strains of* Aspergillus nidulans*, which differ greatly in salt adaption. In total, 2,884 differentially expressed genes including 1,382 up- and 1,502 downregulated genes were identified. Secondary transporters, which were upregulated to a greater extent in Δ*cnaB*Δ*cchA* than in the parent or Δ*cnaB *strains, are likely to play important roles in response to salt stress. Furthermore, 36 genes were exclusively upregulated in the Δ*cnaB*Δ*cchA* under salt stress. Functional analysis of differentially expressed genes revealed that genes involved in transport, heat shock protein binding, and cell division processes were exclusively activated in Δ*cnaB*Δ*cchA*. Overall, our findings reveal that secondary transporters and stress-responsive genes may play crucial roles in salt tolerance to bypass the requirement for the CchA-calcineurin pathway, contributing to a deeper understanding of the mechanisms that influence fungal salt stress adaption in* Aspergillus*.

## 1. Introduction

Rapid adaptation to different environmental conditions, including appropriate adaptive responses to various cations, is crucial for the survival and proliferation of microorganisms. As a closed compartment, the cell possesses intracellular signaling systems that can identify these stimuli in order to implement cellular responses to counteract stress conditions [1]. Calcium signaling is conserved in eukaryotes and plays an important role in sensing environmental stimuli, transmitting extracellular signals to the nucleus to modulate gene expression, regulating morphology, responding to abiotic and biotic stresses, and defending against virulence/pathogenicity [2–4]. Salt stress, one of the major abiotic stresses affecting organismal growth, development, and productivity, can result in damage due to loss of turgor pressure, ion toxicity, membrane disorganization, the generation of reactive oxygen species (ROS), and metabolic toxicity [5, 6]. Hyperosmotic stress caused by NaCl, LiCl, or sorbitol induces an immediate and short duration (1 min) transient cytosolic Ca^2+^ ([Ca^2+^]_cyt_) increase in cells. Small changes in [Ca^2+^]_cyt_ levels can activate various Ca^2+^-sensing proteins, such as calmodulin and calcineurin, which then lead to the induction of various downstream signal transduction pathways [7]. In* Saccharomyces cerevisiae*, the response to salt stress is mediated by calcineurin, a conserved Ca^2+^/calmodulin-modulated protein phosphatase that plays an important role in coupling Ca^2+^ signals to cellular responses. Calcineurin (CaN) is a heterodimer consisting of two subunits, the catalytic subunit A (CnA) and regulatory subunit B (CnB) [8, 9]. Though highly conserved from lower eukaryotes to humans, calcineurin plays diverse and distinct roles in different organisms [10–13]. For instance, in the yeasts* Schizosaccharomyces pombe* and* S. cerevisiae*, it regulates adaptation to a variety of environmental stresses, cation homeostasis, morphogenesis, cell wall integrity, and mating [7, 8, 14–16]. In the pathogenic yeasts* Cryptococcus neoformans* and* Candida albicans*, calcineurin regulates growth at alkaline pH and elevated temperatures and under membrane stress, as well as regulating dimorphism, mating, and virulence [17–19]. In filamentous fungi, it regulates hyphal growth, stress adaptation, and cell wall integrity [20–23].

Organisms employ various regulatory mechanisms for responding to environmental stresses to improve their survival. In yeast, at least two signal transduction pathways are activated to regulate the processes necessary for ion homeostasis and osmotic adjustment. The calcineurin pathway regulates ion homeostasis, while the high osmolarity glycerol (HOG) pathway is primarily responsible for the control of osmotic adaptation [7]. In addition, many other genes, including* NHA1* (encoding a Na^+^/H^+^ antiporter located at the plasma membrane),* TRK1*/*TRK2* (encoding K^+^ transporters located at the plasma membrane), and* NHX1* (encoding a Na^+^/H^+^ antiporter localized in the vacuole), also participate in the salt stress response [24, 25]. Recent studies have indicated that the enzymes implicated in ROS scavenging are crucial for the mitigation of salt-induced oxidative damage [26]. In addition, many heat shock proteins have been extensively identified to improve plant tolerance to salt stress. For example, the overexpression of* HSP18.3*,* OsHsp17.0*, and* OsHsp23.7* confers salt stress tolerance in yeast [27] and rice [28]. In spite of these findings, an integrated and comprehensive dissection of the molecular mechanisms of salt tolerance in* Aspergillus*, in particular, is lacking.

The genus* Aspergillus* consists of several hundreds of mold species that are medically and commercially important and reside in various climates around the world. Among these,* Aspergillus nidulans* is an excellent model organism for filamentous fungi owing to its well-established genetic system, making it easy to study growth, establishment, and maintenance of growth polarity and asexual sporulation. Our previous study revealed that the calcium channel CchA and its regulatory subunit MidA facilitate calcium influx in low-calcium environments and during the stress response in* A. nidulans*. Moreover, the lack of* cchA* in a calcineurin deletion strain (Δ*cnaB*) ameliorated hyphal growth defects induced by salt stress (800 mM NaCl or 600 mM KCl) but not those induced by osmotic stress (1 M sorbitol) [29, 30]. Considering these findings, it is possible that calcineurin-independent mechanisms exist to compensate for the loss of calcineurin-CchA in fungal salt stress adaption.

In the present study, we performed a comprehensive analysis of the transcriptional profiles of genes involved in the salt stress response in* A. nidulans*. We identified candidate genes among differentially expressed genes (DEGs) and carried out a detailed functional analysis of candidate genes and pathways associated with salt tolerance in the parent, Δ*cnaB*, and Δ*cnaB*Δ*cchA* strains. Analysis of DEGs demonstrated that secondary transporters, which were upregulated to a greater extent in Δ*cnaB*Δ*cchA* than in the parent or Δ*cnaB* strains, probably play important roles in salt stress tolerance. Collectively, our findings support previous evidence and indicate that secondary transporters and other stress-responsive genes may participate in a completely unexplored pathway for enhanced salt tolerance. Analysis of such genes should allow for the identification of biomarkers to be used for the establishment of salt tolerance in* A. nidulans*.

## 2. Materials and Methods

### 2.1. Strains, Media, Culture Conditions, and Experimental Groups

A list of* A. nidulans* strains used in this study is given in Table S1 in Supplementary Material available online at https://doi.org/10.1155/2017/4378627. TN02A7, carrying a deletion of a gene required for nonhomologous end joining in double-strand break repair [31], was used as the parent strain. MMPDRUU minimal medium with 2% glucose, nitrate salts, trace elements, 0.5 mg/l pyridoxine, 2.5 mg/l riboflavin, 5 mM uridine, and 10 mM uracil, pH 6.5, was used; trace elements and nitrate salts were added to the medium as described previously [32, 33]. A total of six samples were analyzed: TN02A7, Δ*cnaB,* and Δ*cnaB*Δ*cchA* (Table S1) strains cultured on MMPDRUU comprised groups I (G1), II (G2), and III (G3), respectively, and the same strains cultured on MMPDRUU plus 800 mM NaCl comprised groups IV (G4), V (G5), and VI (G6), respectively.

### 2.2. Total RNA Extraction and Sequencing

For RNA isolation, a total of 1 × 10^8^ conidia from the relevant strains were incubated in 100 ml MMPDRUU at 37°C with shaking at 220 rpm for 18 h. Then, cultures underwent an additional incubation supplemented with or without NaCl (800 mM final concentration) at 37°C for 30 min. The samples were harvested by filtration and ground to a fine powder in liquid nitrogen. Total RNA was isolated according to the method of Simms et al. [34], using TRIzol (Invitrogen, Carlsbad, CA, USA). The RNA was treated with DNase I (amplification grade, Invitrogen) and purified using RNeasy mini kit (Qiagen, Benelux BV, Venlo, The Netherlands) as per the manufacturer's instructions. The final RNA preparations were resuspended in a buffer containing 10 mM Tris hydrochloride, pH 8.0, and 1 mM EDTA. RNA was quantified using a NanoDrop 1000 spectrophotometer v3.7 (NanoDrop Technologies, Inc., Wilmington, DE, USA). cDNA synthesis, library preparation (200 bp inserts), and Illumina sequencing (90 bp paired-end reads) were performed according to the instructions of Trapnell et al. [35] at Oceancloud Gene (Shanghai, China).

### 2.3. Processing and Mapping of Raw Sequences

To obtain high-quality data for analysis, raw reads were cleaned, including removal of reads with adapters. Furthermore, reads with “N” (representative of an ambiguous base) and reads that contained more than 20% bases with* Q* < 20 were discarded. Clean reads were then mapped to the* A. nidulans* genome (assembly ASM14920v1, https://www.ncbi.nlm.nih.gov/genome/17?genome_assembly_id=22574) using TopHat version v2.0.12 [36–38]. Default parameter settings were used for TopHat alignment except “mismatch” which was set to 2. The full data set of this study was deposited in the Gene Expression Omnibus (GEO) with the accession number GSE63019 (https://www.ncbi.nlm.nih.gov/geo/query/acc.cgi?acc=GSE100353).

### 2.4. Analysis of Gene Expression and DEGs

Gene expression analysis was performed via RNA-seq by expectation-maximization (RSEM) [39]. Briefly, the transcript abundance for a given gene was quantified by calculating the frequency of RNA-seq reads aligned to it, and this was expressed as an RPKM value (reads per kilobase of transcript sequence per million mapped reads). Identification of DEGs between two samples was performed using DEGseq [40] after normalizing the read count using TMM [41]. *P* values were adjusted by *q* values, and the threshold for significant differential expression was set at *P* value < 0.001 and |log_2_(fold  change)| > 1.

### 2.5. Enrichment Analysis Using Gene Ontology (GO) and Kyoto Encyclopedia of Genes and Genomes (KEGG)

Functional enrichment of DEGs was determined by GO analysis. GO (http://www.geneontology.org/) is a functional classification system for genes with international standards and is comprised of three ontologies: biological process (BP), cellular component (CC), and molecular function (MF). GO enrichment analysis was performed using GOseq software [42], which is based on Wallenius' noncentral hypergeometric distribution. KEGG (http://www.genome.jp/kegg/) pathway enrichment was performed using KOBAS (2.0) software (http://kobas.cbi.pku.edu.cn/), and DEGs were considered to be significantly enriched in a pathway at a false-discovery rate (FDR) ≤ 0.05.

### 2.6. Verification of the Reliability of RNA-seq by Quantitative Reverse Transcription Polymerase Chain Reaction (qRT-PCR)

Eight genes from among the DEGs were randomly selected for qRT-PCR verification. Gene-specific primers (Table S2) were designed using Primer 5 software, and the actin gene was used as an internal standard. Total RNA isolation was performed as described above. RT-PCR was carried out using HiScript™ Q RT SuperMix (Vazyme, Jiangsu Sheng, China), and the resulting cDNA was used for real-time analysis using SYBR Premix Ex Taq™ (TaKaRa, Tokyo, Japan) as in a previous study [43] on an ABI one-step fast thermocycler as described previously. PCR conditions were as follows: initial denaturation at 95°C for 10 min, followed by 40 cycles of denaturation at 95°C for 5 s, and annealing and extension at 55–58°C for 30 s. Three independent biological replicates were performed, and transcript levels were calculated by the comparative ΔC_T_ method and normalized against the level of actin mRNA. Fold changes in expression were determined by the 2^−ΔΔCT^ method [44].

## 3. Results

### 3.1. Global Analysis of RNA Transcript Data

Approximately 33.3 (G1), 35.6 (G2), 40.7 (G3), 37.6 (G4), 35.7 (G5), and 35.2 (G6) million raw reads were generated, and approximately 28.5 (G1), 30.6 (G2), 35.1 (G3), 32.4 (G4), 30.6 (G5), and 30.2 (G6) million clean reads were obtained, for a total of 218.1 million raw reads and 187.4 million clean reads in this analysis. Clean reads were aligned and mapped against the* A. nidulans* reference genome, resulting in an average of 75.5% of reads mapped (71.4–79.2%). Of the mapped reads, an average of 0.26% (0.2–2.3%) was multiply-mapped to the genome, for a final average of 75.2% (71.2–78.9%) that mapped to unique locations in the genome.

### 3.2. Overall Analysis of DEGs

**Figure 1:**
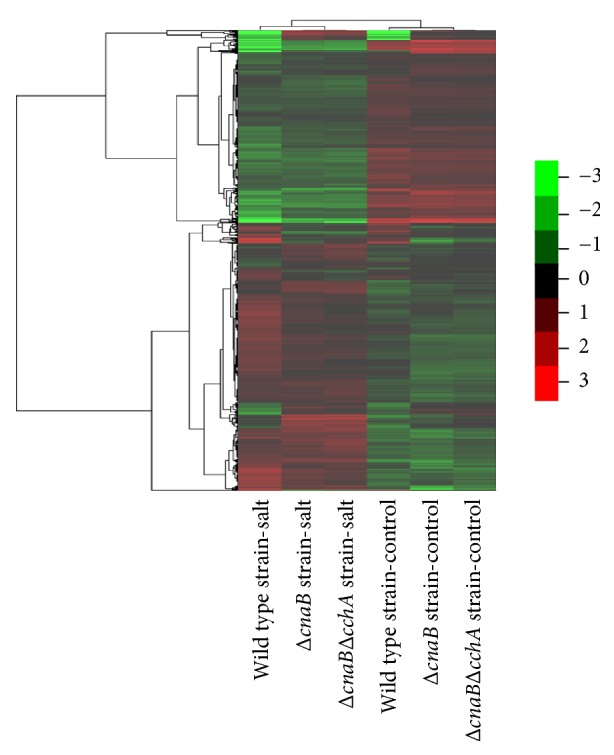
Hierarchical cluster analysis of DEGs. Expression patterns of DEGs in the parent, Δ*cnaB,* and Δ*cnaB*Δ*cchA* strains in the absence and presence of salt stress treatment.

A total of 2,884 genes were identified as DEGs, including 1,382 that were upregulated and 1,502 that were downregulated genes when comparing the mRNA levels of strains under salt stress with those of strains not under salt stress. By comparing the various strains used in the experiment, 1,964 DEGs (1,067 upregulated and 897 downregulated) and 1,863 DEGs (1,016 upregulated and 847 downregulated) were induced by salt stress in Δ*cnaB* and Δ*cnaB*Δ*cchA*, respectively, in comparison to 2,159 DEGs (1,153 upregulated and 1,006 downregulated) induced by salt stress in the parent strain. To characterize patterns of differential gene expression in response to salt stress, a heat map was constructed to visualize the expression patterns of the 2,884 DEGs under different experimental conditions. As shown in Figure 1, DEGs showed different magnitudes of up- and downregulation in response to salt stress in the various strains. Though the DEGs from Δ*cnaB* were similar to those of Δ*cnaB*Δ*cchA*, many genes were identified as being differentially expressed between these two strains. In addition, DEGs clearly self-segregated into salt stress and control clusters.

### 3.3. Shared and Unique DEGs Induced by Salt Stress

**Figure 2:**
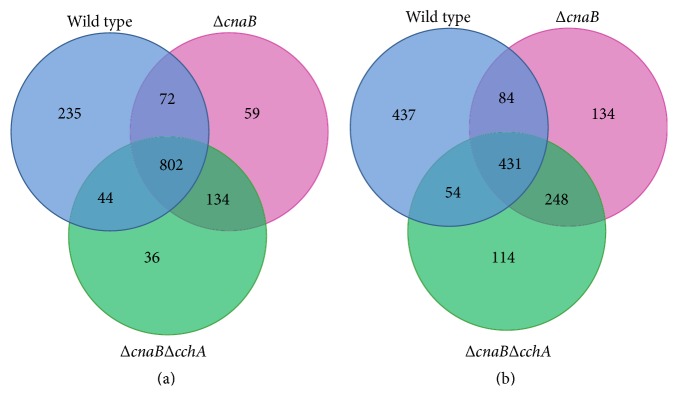
Venn diagram summarizing genes up- or downregulated in each strain under salt stress. DEGs shared by two or three strains are shown in overlapping sections. (a) Numbers of upregulated genes in the parent, Δ*cnaB,* and Δ*cnaB*Δ*cchA* strains under salt stress. (b) Numbers of downregulated genes in the parent, Δ*cnaB,* and Δ*cnaB*Δ*cchA* strains under salt stress.

With the aim of better understanding the shared and unique DEGs induced by salt stress in various strains, we compared changes in gene expression between salt-stressed and nonstressed strains. We used a Venn diagram to reveal DEG distributions after exposure to salt stress in the different strains. A total of 1,382 genes were upregulated in the salt stress strains when compared with expression levels in the control strain, including 802 genes (58.03%) that were upregulated in all three salt-treated strains. Interestingly, fewer genes were exclusively upregulated in Δ*cnaB*Δ*cchA* (36) than in the parent (235) or Δ*cnaB* (59) strains under salt stress (Figure 2(a)). Moreover, 1,502 genes were downregulated, among which only 431 were shared among all strains, and the numbers of genes that were exclusively downregulated in the parent, Δ*cnaB,* and Δ*cnaB*Δ*cchA* strains were 437, 134, and 114, respectively (Figure 2(b)). Interestingly, the largest number of DEGs was observed in the parent strain, while the fewest were observed in Δ*cnaB*Δ*cchA*.

### 3.4. Identification of Candidate Genes for Salt Tolerance in Δ*cnaB*Δ*cchA*

**Figure 3:**
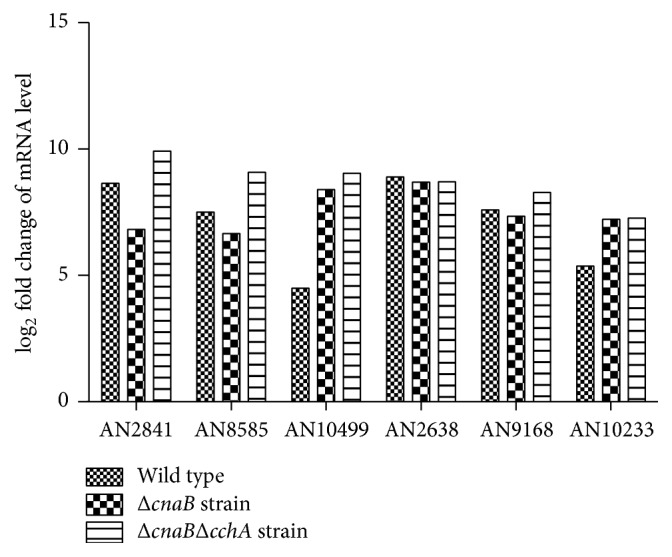
Relative upregulation in mRNA levels of secondary transporter genes under salt stress. Fold changes in mRNA levels of* AN2841* (MFS_1 major facilitator superfamily),* AN8585* (Rossmann-fold NAD(P)(+)-binding proteins),* AN10499* (aldo/ketoreductase, related to diketogulonate reductase, secondary metabolite biosynthesis, transport, and catabolism),* AN2638* (hexokinase),* AN9168* (conserved hypothetical protein-putative sugar transporter), and* AN3210* (MFS myo-inositol transporter) after salt stress treatment.

Since our previously published data showed that the deletion of* cchA* could suppress hyphal growth defects caused by the loss of calcineurin under salt stress in* A. nidulans* [30], we sought to identify the genes responsible for the restoration of hyphal growth in Δ*cnaB*Δ*cchA*. Candidate genes for hyphal growth remediation were those that were upregulated to a greater extent in Δ*cnaB*Δ*cchA* than in the other strains and that were exclusively induced in Δ*cnaB*Δ*cchA*. It is noteworthy that secondary transporter genes, including* AN2841* (MFS_1 major facilitator superfamily),* AN8585* (Rossmann-fold NAD(P)(+)-binding proteins),* AN10499* (secondary metabolite biosynthesis, transport, and catabolism),* AN2638* (hexokinase),* AN9168* (MFS, conserved hypothetical protein-putative sugar transporter), and* AN3210* (MFS myo-inositol transporter), were significantly upregulated more than 4.5-fold under salt stress in all strains. However, the above secondary transporters were upregulated to a greater extent in Δ*cnaB*Δ*cchA* than in the other strains. As shown in Figure 3, in Δ*cnaB*Δ*cchA*, expression levels of* AN2841*,* AN8585,* and* AN9168* were upregulated 10.0-, 8.5-, and 8.2-fold, respectively, in response to salt stress while they were upregulated only 7.0-, 6.8-, and 7.1-fold, respectively, in Δ*cnaB*. As expected, the above secondary transporter genes were among the top 12 genes with the greatest upregulation in Δ*cnaB*Δ*cchA*. Based on this, these transporter genes were selected as candidate genes for both salt tolerance and hyphal growth remediation. Specifically, among the 36 upregulated genes unique to Δ*cnaB*Δ*cchA*,* AN0531* (secondary transporter* AcrB*),* AN5593* (F-box and WD domain protein),* AN6718* (AAA family ATPase),* AN8035* (heat shock transcription factor), and* AN9528* (maintenance of mitochondrial morphology protein 1, MMM1) have been reported to be activated by abiotic stress in other organisms [45–51]. Hence, it seems that the upregulation of secondary transporters and increased expression of the above DEGs in Δ*cnaB*Δ*cchA* may lead to hyphal growth remediation in response to salt stress.

### 3.5. Validation of RNA-seq Results by qRT-PCR

**Figure 4:**
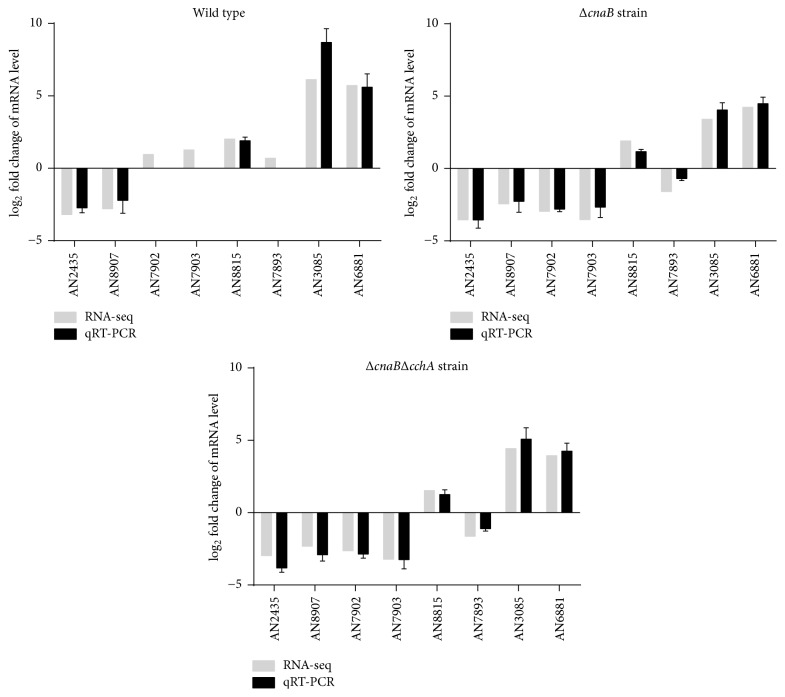
Validation of RNA-seq data by qRT-PCR. Eight genes were randomly selected from the entire genome, and expression levels were measured by qRT-PCR in the relevant strains in the presence and absence of salt stress treatment.

To validate the reliability of our transcriptome sequencing data, eight DEGs were randomly selected for qRT-PCR validation in the relevant strains in the presence or absence of 800 mM NaCl. Fold changes from qRT-PCR were compared with the RNA-seq expression analysis results. As expected, the results of qRT-PCR were similar to those of RNA-seq, with virtually identical trends in up- or downregulation for each gene in the indicated strains (Figure 4), suggesting the reliability and accuracy of the RNA-seq expression analysis.

### 3.6. Gene Ontology Analysis of DEGs

**Figure 5:**
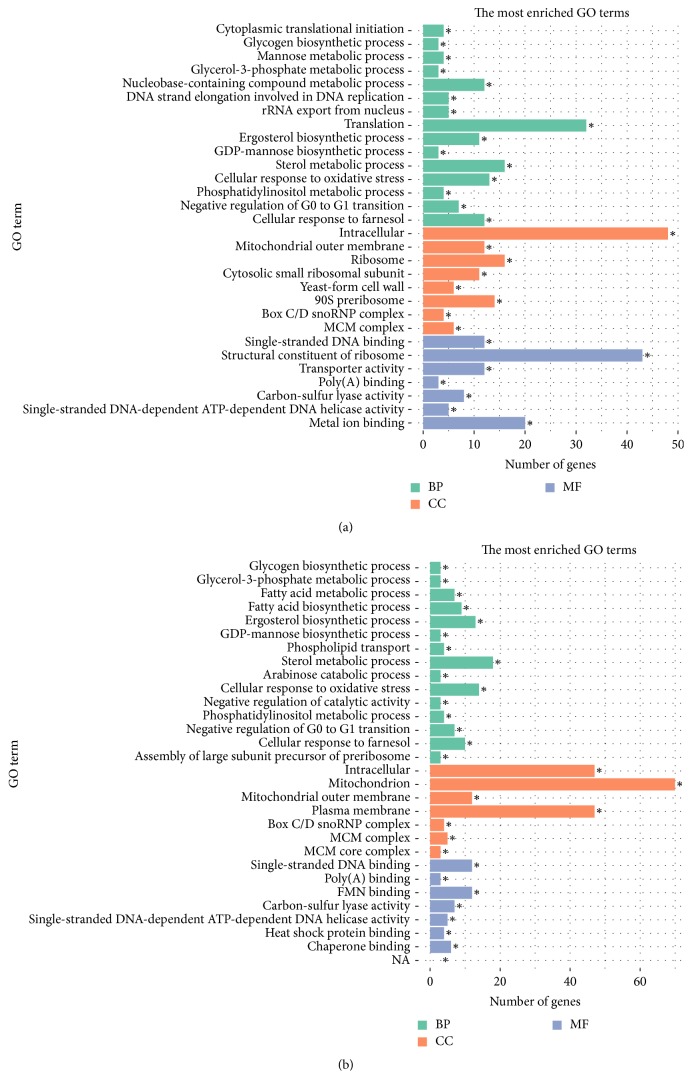
Most enriched GO terms among DEGs in Δ*cnaB* and Δ*cnaB*Δ*cchA* strains. (a) Most enriched GO terms among DEGs induced by salt stress in Δ*cnaB*. (b) Most enriched GO terms among DEGs induced by salt stress in Δ*cnaB*Δ*cchA*. For each enriched GO term with a Bonferroni *P* value < 0.05, the ontology to which the GO term belongs is shown (BP = biological process; CC = cellular component; MF = molecular function). ^*∗*^*p* < 0.05.

To gain insight into the functional roles of DEGs in the regulation of fungal hyphal growth under salt stress, we conducted GO enrichment analysis of DEGs using GOseq software. For GO term analysis, up- and downregulated DEGs were annotated with the biological process, molecular function, and cellular component ontologies. Of the 30 most enriched GO terms, 17 were shared by Δ*cnaB* and Δ*cnaB*Δ*cchA*, such as the terms cellular response to oxidative stress, negative regulation of G0 to G1 transition, mitochondrial outer membrane, intracellular, glycerol-3-phosphate metabolic process, and phosphatidylinositol metabolic process (Figures 5(a) and 5(b)). In particular, 12 significantly enriched GO terms, such as those related to transport processes (i.e., the GO terms transport, Golgi to plasma membrane protein transport, and P-P-bond-hydrolysis-driven protein transmembrane transporter activity), protein binding processes (heat shock protein binding and proteasome binding), and cell growth processes (mitotic spindle elongation and spindle midzone), were associated with response to salt stress exclusively in Δ*cnaB*Δ*cchA* (Table S3). As shown in Figure S1, the most enriched GO terms in response to salt stress in the parent strain were translation, nucleolus, cytosol, ribosome associative function, RNA binding, and RNA polymerase activity (RNA polymerase I and RNA polymerase III). In addition, GO terms associated with calcium channels, such as calcium ion transport, cellular calcium ion homeostasis, sulfate transport, cellular iron ion homeostasis, cellular response to osmotic stress, nucleocytoplasmic transport, protein channel activity, and electron carrier activity, were exclusively enriched in the parent strain (Table S4), further indicating that the calcium signaling pathway plays a vital role in the adaptation of fungi to salt stress by regulating the influx and efflux of ions.

### 3.7. KEGG Classifications of DEGs

**Figure 6:**
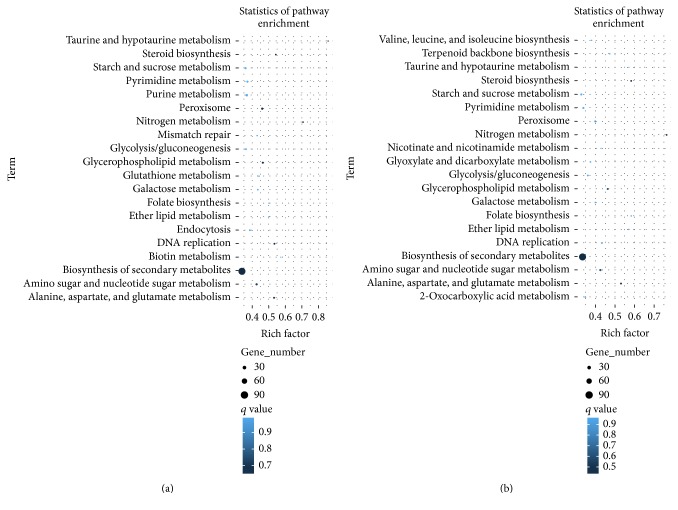
KEGG pathway enrichment analysis of DEGs in Δ*cnaB* and Δ*cnaB*Δ*cchA* strains. (a) KEGG pathway enrichment of DEGs in Δ*cnaB*. (b) KEGG pathway enrichment of DEGs in Δ*cnaB*Δ*cchA*. The *x*-axis indicates the enrichment factor of each pathway, and the *y*-axis indicates each pathway.

To gain insight into the physiological and gene pathways involved in the response to salt stress in each strain, DEGs were classified using the KEGG database (Figures 6(a) and 6(b) and S2). This database integrates genomic, chemical, and systemic functional information, aiding in the study of the complex biological behaviors of genes. KEGG classification of genes differentially expressed in strains under salt stress revealed that the RNA polymerase, ribosome biogenesis in eukaryotes, and vitamin B6 metabolism pathways were the most enriched in the parent strain (Figure S2), while the taurine and hypotaurine metabolism, nitrogen metabolism, and steroid biosynthesis pathways were the most enriched in Δ*cnaB* (Figure 6(a)). In Δ*cnaB*Δ*cchA* under salt stress, the most enriched pathways were nitrogen metabolism, followed by steroid biosynthesis and folate biosynthesis (Figure 6(b)).

## 4. Discussion

**Figure 7:**
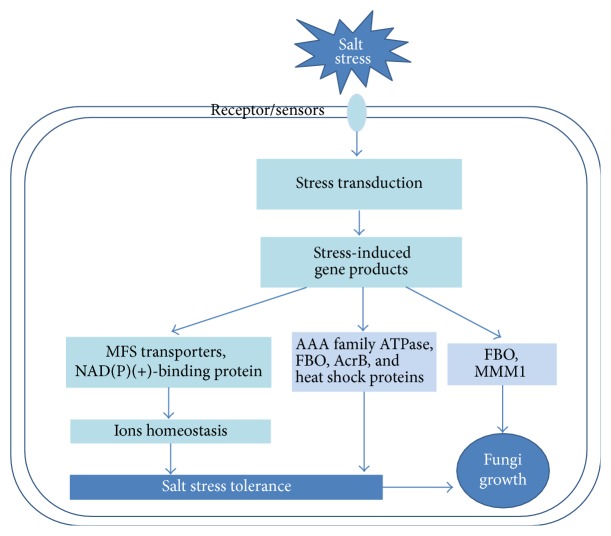
Signal transduction network of salt stress response and growth in* A. nidulans.*

Our previously published data showed that the deletion of* cchA* could ameliorate hyphal growth defects caused by the loss of calcineurin under salt stress in* A. nidulans*. A number of studies have highlighted the importance of calcineurin in the stress response in both fungi [11, 14, 19, 23]; however, the complex molecular responses and signal transduction pathways that may compensate for the function of calcineurin-CchA in fungi under salt stress remain to be fully elucidated. RNA-seq analyses of transcriptomic profiles are commonly used as a robust approach for assessing transcriptional responses to different experimental conditions [52, 53]. Therefore, we explored global expression differences among the parent, Δ*cnaB*, and Δ*cnaB*Δ*cchA* strains of* A. nidulans* in response to salt stress in order to identify candidate genes that mediate salt tolerance in a calcineurin/CchA-independent manner. From the results, we developed a tentative model for the signal tranduction network of salt stress responses and growth in* A. nidulans* (Figure 7). The keys to understand the potential mechanisms of salt stress adaption in* A. nidulans* are discussed below.

Emerging data have shown that secondary metabolism is closely related to the regulation of the oxidative stress response in fungi. In* A. nidulans*, Emri et al. identified that various stresses, including NaCl, significantly influence the synthesis of secondary metabolites, as they affect the transcription of secondary metabolite biosynthetic loci [54]. Similarly, our transcriptome data also indicated that the GO term “Biosynthesis of secondary metabolites” was enriched in both the parent and the deletion strains (data not shown). Moreover, previous studies have indicated that* AtfA* is essential for the tolerance to oxidative and heat stress, as well as normal vegetative growth and sporulation in* A. nidulans* [55–57]. In addition, numerous genes are regulated through the SskA–HogA–AtfA signaling pathway in response to nonionic osmotic stress in* A. nidulans* triggered by sorbitol [55]. Our study found that the MAPK pathway was typical of the upregulated genes and that the expression of* hogA* and* atfA* was upregulated in all strains under unstressed conditions, indicating that the salt stress regulation of* atfA* was calcium channel-independent. In the present study, the parent strain demonstrated a transcriptional upregulation of genes encoding proteins involved in carbohydrate, lipid, and trehalose metabolic processes, while genes encoding proteins involved in primary metabolic processes, RNA processing, and DNA-dependent DNA replication initiation were downregulated, which is in accordance with the results for* Aspergillus fumigatus* under osmotic stress [58].

To date, it has been revealed that secondary transporters, including uniporters, symporters, and antiporters, facilitate tolerance to salt stress through the transportation of a number of ions and secondary metabolic substrates across cytoplasmic or internal membranes in a variety of organisms. The MFS are a primary class of uniporters, symporters, and antiporters [59, 60]. These proteins play vital roles in ion homeostasis, such as Na^+^ uptake via phosphate, potassium, and hydrogen ions under salt stress [60, 61]. In the rat, uniporters have been reported to play an important role in maintaining cell permeability [62]. Moreover, symporters are major determinants in the resistance of* Mesembryanthemum crystallinum* to salt stress [63]. Studies in adult* Drosophila* have shown that when exposed to dietary salt stress, genes encoding symporters were significantly overexpressed. Furthermore, the critical roles of antiporters in salt stress resistance have been widely reported. For instance, a study in* Drosophila* and humans revealed that Nha1 and Nha2, members of the cation/proton antiporter (CPA) family, are essential for ion homeostasis and salt stress [64]. Similarly, a recent study indicated that TaNHX3, a Na^+^/H^+^ antiporter gene, plays an important role in regulating cytosolic Na^+^ transport within vacuoles under high salinity, alleviating the damaging effects of Na^+^; overexpression of TaNHX3 enhances salt tolerance in tobacco plants [65].

Consistent with previous reports, our data indicate that the mRNA levels of secondary transporter genes are significantly increased in response to salt stress. The expression levels of* AN2841*,* AN8585*, and* AN9168* were upregulated to a greater extent in Δ*cnaB*Δ*cchA* than in Δ*cnaB* in response to salt stress. With the exception of* AN2638*, all secondary transporter genes were upregulated to a greater extent in Δ*cnaB*Δ*cchA* than in the parent and Δ*cnaB* strains, suggesting that secondary transporters may help cells to avoid the toxicity of harmful ions via the transport system. These transporters likely play a critical role in improving salt stress tolerance in fungi, thus bypassing the requirement for calcineurin to some extent during salt stress in Δ*cnaB*Δ*cchA*.

Among the 36 genes exclusively induced in Δ*cnaB*Δ*cchA*, the increased expression of six responsive genes in particular, transporter* AcrB*, AAA family ATPase, F-box and WD protein* (FBO)*, heat shock protein, and maintenance of mitochondrial morphology protein 1* (MMM1)*, has been reported to be activated by abiotic stress in other organisms and may lead to hyphal growth remediation and salt adaption in response to high NaCl stress in Δ*cnaB*Δ*cchA*. For example, the multidrug transporter AcrB, a heavy metal cation efflux protein, plays a significant role in salt resistance in* Escherichia coli* [45]. According to previous reports [46], AAA family ATPase contributes to shoot adaption to salt stress in Tibetan wild barley. In addition, previous research on the fungus* Ascochyta rabiei* found that expression of F-box and WD protein* (FBO)* and several heat shock proteins was highly induced under oxidative stress [47]. Moreover, FBO proteins have been shown to act as scavenger proteins in the cell [48] and to control the cell division cycle, morphogenesis, nutrient sensing, and calcium signaling in various fungi, such as* Magnaporthe oryzae* and* Fusarium oxysporum* [49]. Furthermore, F-box domain proteins have been reported to play crucial roles in regulating various developmental processes and stress responses [51]. A study in* A. fumigatus* showed that the MMM1 protein is important in maintaining mitochondrial morphology; the ER–mitochondria structure accelerates hyphal growth and is involved in virulence of the pathogenic mold [50]. Therefore, it is probable that FBO and MMM1 have an active influence on growth and development in fungi. Taken together, these findings suggest that the increased expression of the above genes in Δ*cnaB*Δ*cchA* may mediate salt adaptation in a calcineurin/CchA-independent manner in response to salt stress.

Filamentous fungi grow through elongating hyphae at their tips to generate primary hyphae that branch to produce secondary hyphae [66]. This involves cytoplasmic streaming to the apical tip, which occurs by the establishment of internal osmotic gradients involving the transport of various ions toward the hyphae [67]. As expected, in the present study, the functional classification of DEGs in Δ*cnaB*Δ*cchA* showed that transport processes (transport, Golgi to plasma membrane protein transport, and P-P-bond-hydrolysis-driven protein transmembrane transporter activity), heat shock protein binding, and cell growth processes (mitotic spindle elongation and spindle midzone) were specifically induced by salt stress, while the parent strain was exclusively enriched for functions related to calcium, indicating that cellular transportation mechanisms compensate for the loss of calcineurin-CchA and play pivotal roles in fungal hyphal growth under salt stress. Spindle elongation and spindle midzone processes play important roles in mitosis, and previous studies from our laboratory and from others have consistently shown that timely cytokinesis/septation (cell division) is essential for hyphal growth in filamentous fungi [68, 69]. Consequently, these results imply that transporter genes represent a potentially unexplored pathway for enhancing salt tolerance. Genes related to transport, heat shock, and mitosis may play roles in the restoration of hyphal growth in Δ*cnaB*Δ*cchA* under salt stress.

In summary, this work identified the roles of secondary transporters and other stress-responsive genes in the abiotic salt stress response, providing the basis for a calcineurin-CchA-independent mechanism of salt stress adaption in* Aspergillus*. The candidate genes identified here should be valuable for further targeted studies on salt tolerance in fungi. However, more work is needed to further characterize the products of these calcineurin-independent genes and to elucidate their roles in the response to environmental stress in* Aspergillus*. Notably, more detailed knowledge of the genes involved in salt tolerance in fungi, together with a greater understanding of the mechanisms underlying the development of fungal polarized growth, will be of great help in finding novel drug therapies for fungal infections.

## Supplementary Material

Table S1. List of A. nidulans strains used in this study. Table S2. List of primers used in this study. Table S3. Unique significant enriched GO terms related to ΔcnaBΔcchA strain following salt stress challenge. Table S4. Unique significant enriched GO terms involved in wild type strain following salt stress challenge. Figure S1. Most enriched GO terms among DEGs induced by salt stress in wild-type A. nidulans. For each enriched GO term with a Bonferroni P-value < 0.01, the ontology to which the GO term belongs is shown (BP = Biological Process; CC = Cellular Component; MF = Molecular Function). Figure S2. KEGG pathway enrichment of DEGs induced by salt stress in wild-type A. nidulans. The x-axis indicates the enrichment factor of each pathway, and the y-axis indicates each pathway.

## References

[1] Colinet A.-S., Sengottaiyan P., Deschamps A. (2016). Yeast Gdt1 is a Golgi-localized calcium transporter required for stress-induced calcium signaling and protein glycosylation. *Scientific Reports*.

[2] Miskei M., Karányi Z., Pócsi I. (2009). Annotation of stress-response proteins in the aspergilli.. *Fungal genetics and biology : FG & B*.

[3] Saito H. (2010). Regulation of cross-talk in yeast MAPK signaling pathways. *Current Opinion in Microbiology*.

[4] Xu J.-R. (2000). MAP kinases in fungal pathogens. *Fungal Genetics and Biology*.

[5] Zhang J.-L., Shi H. (2013). Physiological and molecular mechanisms of plant salt tolerance. *Photosynthesis Research*.

[6] Parida A., Das A. B., Das P. (2002). NaCl stress causes changes in photosynthetic pigments, proteins, and other metabolic components in the leaves of a true mangrove,Bruguiera parviflora, in hydroponic cultures. *Journal of Plant Biology*.

[7] Matsumoto T. K., Ellsmore A. J., Cessna S. G. (2002). An osmotically induced cytosolic Ca2+ transient activates calcineurin signaling to mediate ion homeostasis and salt tolerance of Saccharomyces cerevisiae. *Journal of Biological Chemistry*.

[8] Mendoza I., Rubio F., Rodriguez-Navarro A., Pardo J. M. (1994). The protein phosphatase calcineurin is essential for NaCl tolerance of Saccharomyces cerevisiae. *Journal of Biological Chemistry*.

[9] Rusnak F., Mertz P. (2000). Calcineurin: form and function. *Physiological Reviews*.

[10] Fox D. S., Cruz M. C., Sia R. A. L. (2001). Calcineurin regulatory subunit is essential for virulence and mediates interactions with FKBP12-FK506 in Cryptococcus neoformans. *Molecular Microbiology*.

[11] Juvvadi P. R., Lamoth F., Steinbach W. J. (2014). Calcineurin as a multifunctional regulator: unraveling novel functions in fungal stress responses, hyphal growth, drug resistance, and pathogenesis. *Fungal Biology Reviews*.

[12] Steinbach W. J., Cramer R. A., Perfect B. Z. (2006). Calcineurin controls growth, morphology, and pathogenicity in Aspergillus fumigatus. *Eukaryotic Cell*.

[13] Stie J., Fox D. (2008). Calcineurin regulation in fungi and beyond. *Eukaryotic Cell*.

[14] Cyert M. S. (2003). Calcineurin signaling in *Saccharomyces cerevisiae*: how yeast go crazy in response to stress. *Biochemical and Biophysical Research Communications*.

[15] Mendoza I., Quintero F. J., Bressan R. A., Hasegawa P. M., Pardo J. M. (1996). Activated calcineurin confers high tolerance to ion stress and alters the budding pattern and cell morphology of yeast cells. *Journal of Biological Chemistry*.

[16] Yoshida T., Toda T., Yanagida M. (1994). A calcineurin-like gene ppb1+ in fission yeast: Mutant defects in cytokinesis, cell polarity, mating and spindle pole body positioning. *Journal of Cell Science*.

[17] Sanglard D., Ischer F., Marchetti O., Entenza J., Bille J. (2003). Calcineurin A of Candida albicans: Involvement in antifungal tolerance, cell morphogenesis and virulence. *Molecular Microbiology*.

[18] Cruz M. C., Fox D. S., Heitman J. (2001). Calcineurin is required for hyphal elongation during mating and haploid fruiting in Cryptococcus neoformans. *EMBO Journal*.

[19] Cruz M. C., Goldstein A. L. (2002). Calcineurin is essential for survival during membrane stress in Candida albicans. *EMBO Journal*.

[20] Kothe G. O., Free S. J. (1998). Calcineurin subunit B is required for normal vegetative growth in Neurospora crassa. *Fungal Genetics and Biology*.

[21] Nanthakumar N. N., Dayton J. S., Means A. R. (1996). Role of Ca++/calmodulin binding proteins in Aspergillus nidulans cell cycle regulation. *Prog Cell Cycle Res*.

[22] Harel A., Bercovich S., Yarden O. (2006). Calcineurin is required for sclerotial development and pathogenicity of Sclerotinia Sclerotiorum in an oxalic acid-independent manner. *Molecular Plant-Microbe Interactions*.

[23] Cervantes-Chávez J. A., Ali S., Bakkeren G. (2011). Response to environmental stresses, cell-wall integrity, and virulence are orchestrated through the calcineurin pathway in ustilago hordei. *Molecular Plant-Microbe Interactions*.

[24] Serrano R., Mulet J. M., Rios G. (1999). A glimpse of the mechanisms of ion homeostasis during salt stress. *Journal of Experimental Botany*.

[25] Serrano R., Gaxiola R. (1994). Microbial models and salt stress tolerance in plants. *Critical Reviews in Plant Sciences*.

[26] Naliwajski M. R., Skłodowska M. (2014). The oxidative stress and antioxidant systems in cucumber cells during acclimation to salinity. *Biologia Plantarum*.

[27] Gao C., Jiang B., Wang Y., Liu G., Yang C. (2012). Overexpression of a heat shock protein (ThHSP18.3) from Tamarix hispida confers stress tolerance to yeast. *Molecular Biology Reports*.

[28] Zou J., Liu C., Liu A., Zou D., Chen X. (2012). Overexpression of OsHsp17.0 and OsHsp23.7 enhances drought and salt tolerance in rice. *Journal of Plant Physiology*.

[29] Wang S., Cao J., Liu X. (2012). Putative calcium channels CchA and MidA play the important roles in conidiation, hyphal polarity and cell wall components in *Aspergillus nidulans*. *PLoS ONE*.

[30] Wang S., Liu X., Qian H., Zhang S., Lu L. (2016). Calcineurin and calcium channel CchA coordinate the salt stress response by regulating cytoplasmic Ca2+ homeostasis in Aspergillus nidulans. *Applied and Environmental Microbiology*.

[31] Nayak T., Szewczyk E., Oakley C. E. (2006). A versatile and efficient gene-targeting system for Aspergillus nidulans. *Genetics*.

[32] Käfer E. (1977). Meiotic and Mitotic Recombination in Aspergillus and Its Chromosomal Aberrations. *Advances in Genetics*.

[33] Wang J., Hu H., Wang S. (2009). The important role of actinin-like protein (AcnA) in cytokinesis and apical dominance of hyphal cells in Aspergillus nidulans. *Microbiology*.

[34] Simms D., Cizdziel P. E., Chomczynski P. (1993). TRIzol: a new reagent for optimal single-step isolation of RNA. *Focus*.

[35] Trapnell C., Williams B. A., Pertea G. (2010). Transcript assembly and quantification by RNA-Seq reveals unannotated transcripts and isoform switching during cell differentiation. *Nature Biotechnology*.

[36] Brueffer C., Saal L. H. (2016). TopHat-Recondition: a post-processor for TopHat unmapped reads. *BMC Bioinformatics*.

[37] Ghosh S., Chan C. K. (2016). Analysis of RNA-seq data using TopHat and cufflinks. *Plant Bioinformatics: Methods and Protocols*.

[38] Pollier J., Rombauts S., Goossens A. (2013). Analysis of RNA-Seq data with TopHat and cufflinks for genome-wide expression analysis of jasmonate-treated plants and plant cultures. *Methods in Molecular Biology*.

[39] Li B., Dewey C. N. (2011). RSEM: accurate transcript quantification from RNA-Seq data with or without a reference genome. *BMC Bioinformatics*.

[40] Wang L., Feng Z., Wang X., Zhang X. (2010). DEGseq: an R package for identifying differentially expressed genes from RNA-seq data. *Bioinformatics*.

[41] Robinson M. D., Oshlack A. (2010). A scaling normalization method for differential expression analysis of RNA-seq data. *Genome Biology*.

[42] Anders S., Huber W. (2010). Differential expression analysis for sequence count data. *Genome Biology*.

[43] Ni M., Yu J.-H. (2007). A novel regulator couples sporogenesis and trehalose biogenesis in aspergillus nidulans. *PLoS ONE*.

[44] Livak K. J., Schmittgen T. D. (2001). Analysis of relative gene expression data using real-time quantitative PCR and the 2(-Delta Delta C(T)) method. *Methods*.

[45] Paul S., Alegre K. O., Holdsworth S. R. (2014). A single-component multidrug transporter of the major facilitator superfamily is part of a network that protects Escherichia coli from bile salt stress. *Molecular Microbiology*.

[46] Shen Q., Fu L., Dai F., Jiang L., Zhang G., Wu D. (2016). Multi-omics analysis reveals molecular mechanisms of shoot adaption to salt stress in Tibetan wild barley. *BMC Genomics*.

[47] Singh K., Nizam S., Sinha M., Verma P. K. (2012). Comparative transcriptome analysis of the necrotrophic fungus Ascochyta rabiei during oxidative stress: Insight for fungal survival in the host plant. *PLoS ONE*.

[48] Skowyra D., Craig K. L., Tyers M., Elledge S. J., Harper J. W. (1997). F-box proteins are receptors that recruit phosphorylated substrates to the SCF ubiquitin-ligase complex. *Cell*.

[49] Jonkers W., Rep M. (2009). Lessons from fungal F-Box proteins. *Eukaryotic Cell*.

[50] Geißel B., Penka M., Neubauer M., Wagener J. (2017). The ER-mitochondria encounter structure contributes to hyphal growth, mitochondrial morphology and virulence of the pathogenic mold Aspergillus fumigatus. *International Journal of Medical Microbiology*.

[51] Yan Y.-S., Chen X.-Y., Yang K. (2011). Overexpression of an F-box protein gene reduces abiotic stress tolerance and promotes root growth in rice. *Molecular Plant*.

[52] Nookaew I., Papini M., Pornputtapong N. (2012). A comprehensive comparison of RNA-Seq-based transcriptome analysis from reads to differential gene expression and cross-comparison with microarrays: A case study in Saccharomyces cerevisiae. *Nucleic Acids Research*.

[53] Oshlack A., Robinson M. D., Young M. D. (2010). From RNA-seq reads to differential expression results. *Genome Biology*.

[54] Emri T., Szarvas V., Orosz E. (2015). Core oxidative stress response in Aspergillus nidulans. *BMC Genomics*.

[55] Hagiwara D., Asano Y., Marui J., Yoshimi A., Mizuno T., Abe K. (2009). Transcriptional profiling for Aspergillus nidulans HogA MAPK signaling pathway in response to fludioxonil and osmotic stress. *Fungal Genetics and Biology*.

[56] Hagiwara D., Asano Y., Yamashino T., Mizuno T. (2008). Characterization of bZip-type transcription factor AtfA with reference to stress responses of conidia of Aspergillus nidulans. *Bioscience, Biotechnology and Biochemistry*.

[57] Lara-Rojas F., Sánchez O., Kawasaki L., Aguirre J. (2011). Aspergillus nidulans transcription factor AtfA interacts with the MAPK SakA to regulate general stress responses, development and spore functions. *Molecular Microbiology*.

[58] Pereira Silva L., Alves de Castro P., dos Reis T. F. (2017). Genome-wide transcriptome analysis of Aspergillus fumigatus exposed to osmotic stress reveals regulators of osmotic and cell wall stresses that are SakAHOG1 and MpkC dependent. *Cellular Microbiology*.

[59] Marger M. D., Saier M. H. (1993). A major superfamily of transmembrane facilitators that catalyse uniport, symport and antiport. *Trends in Biochemical Sciences*.

[60] Sekhwal M. K., Sharma V., Sarin R. (2013). Identification of MFS proteins in sorghum using semantic similarity. *Theory in Biosciences*.

[61] Tester M., Davenport R. (2003). Na^+^ tolerance and Na^+^ transport in higher plants. *Annals of Botany*.

[62] Lapidus R. G., Sokolove P. M. (1993). Spermine Inhibition of the Permeability Transition of Isolated Rat Liver Mitochondria: An Investigation of Mechanism. *Archives of Biochemistry and Biophysics*.

[63] Chauhan S., Forsthoefel N., Ran Y., Quigley F., Nelson D. E., Bohnert H. J. (2000). Na+/myo-inositol symporters and Na+/H+-antiport in Mesembryanthemum crystallinum. *Plant Journal*.

[64] Chintapalli V. R., Kato A., Henderson L. (2015). Transport proteins NHA1 and NHA2 are essential for survival, but have distinct transport modalities. *Proceedings of the National Academy of Sciences of the United States of America*.

[65] Lu W., Guo C., Li X. (2014). Overexpression of TaNHX3, a vacuolar Na+/H+ antiporter gene inwheat, enhances salt stress tolerance in tobacco by improving related physiological processes. *Plant Physiology and Biochemistry*.

[66] Knoblach B., Rachubinski R. A. (2016). How peroxisomes partition between cells. A story of yeast, mammals and filamentous fungi. *Current Opinion in Cell Biology*.

[67] Lew R. R. (2005). Mass flow and pressure-driven hyphal extension in Neurospora crassa. *Microbiology*.

[68] Inoue S. (1981). Cell division and the mitotic spindle. *The Journal of Cell Biology*.

[69] Zhong G., Wei W., Guan Q. (2012). Phosphoribosyl pyrophosphate synthetase, as a suppressor of the seph mutation in Aspergillus nidulans, is required for the proper timing of septation. *Molecular Microbiology*.

