# Supplementary material for: Transcriptomic Analysis Reveals Genes Mediating Salt Tolerance through Calcineurin/CchA-Independent Signaling in Aspergillus nidulans
Source: Biomed Res Int. 2017 Aug 20;2017:4378627. doi: 10.1155/2017/4378627 (PMC5585587; doi:10.1155/2017/4378627)
Supplement: Supplementary file 1 — Table S1. List of A. nidulans strains used in this study. Table S2. List of primers used in this study. Table S3. Unique significant enriched GO terms related to ΔcnaBΔcchA strain following salt stress challenge. Table S4. Unique significant enriched GO terms involved in wild type strain following salt stress challenge. Figure S1. Most enriched GO terms among DEGs induced by salt stress in wild-type A. nidulans. For each enriched GO term with a Bonferroni P-value < 0.01, the ontology to which the GO term belongs is shown (BP = Biological Process; CC = Cellular Component; MF = Molecular Function). Figure S2. KEGG pathway enrichment of DEGs induced by salt stress in wild-type A. nidulans. The x-axis indicates the enrichment factor of each pathway, and the y-axis indicates each pathway. [file 4378627.f1.docx]

**TABLE S1** List of *A. nidulans* strains used in this study

| Strain | Genotype | | Reference  or source |
| --- | --- | --- | --- |
| TN02A7 | | *pyrG89*; *riboB2*; *pyroA4 nkuA*::*argB2*; *veA1* | Nayak et al. (2006) |
| WSA05 | | *pyrG89*; *riboB2*; *pyroA4 nkuA*::*argB2*; Δ*cchA*::*pyrG*; *veA1* | Wang et al. (2012) |
| WSA08 | | *pyrG8*9 Δ*cnaB*::*pyrG*; *riboB2*; *pyroA4nkuA*::*argB2*; *veA1* | Wang et al. (2016) |
| LXA05 | | *pyrG89* Δ*cnaB*::*pyrG*; *nkuA*::*argB2*;Δ*cchA*::*pyrG*; *veA1* | Wang et al. (2016) |

**TABLE S2** List of primers used in this study

| Primer | DNA sequence (5'-3') |
| --- | --- |
| actin-RT-up | TCTTCCAGCCCAGCGTTCT |
| actin-RT-down | GGGCGGTGATTTCCTTCTG |
| AN2435- RT-up | GAGCAAGGAGGAGAAGTTC |
| AN2435- RT-down | GCAATACCACCACCGATAA |
| AN8907- RT-up | ATCCGATGATGGAAGTCTG |
| AN8907- RT-down | CGTATTGGTGGTGAATGC |
| AN7902- RT-up | TACAAGTCGCCATCATAGG |
| AN7902- RT-down | ATCCATCGCAGATAGTCATT |
| AN7903- RT-up | GCCTGCGACTATGAGAATA |
| AN7903- RT-down | CACCTCTACAGTAGCCATC |
| 8AN8815- RT-up | GGTGTCAAGCGATTCATTC |
| 8AN8815- RT-down | CGGTGTTGTATGCGAAGA |
| 9AN7893- RT-up | GATTCCTGAGCCTCGTAG |
| 9AN7893- RT-down | TAATGAAGCACTCCTTGGTA |
| 10AN3085- RT-up | TATCGCCGCTTGTCTAAC |
| 10AN3085- RT-down | TCATCTGTCGTGGTGGTA |
| 11AN6881- RT-up | CGCCACTTCTACGGAATT |
| 11AN6881- RT-down | AGGTTATTCGCATCGTCTT |

**TABLE S3** Unique significant enriched GO terms related to Δ*cnaB*Δ*cchA* strain following salt stress challenge

| category | over_represented_pvalue | numDEInCat | term |
| --- | --- | --- | --- |
| GO:0006631 | 0.014486 | 7 | fatty acid metabolic process |
| GO:0008150 | 0.021508 | 780 | biological_process |
| GO:0019287 | 0.027874 | 3 | isopentenyl diphosphate biosynthetic process, mevalonate pathway |
| GO:0006415 | 0.03318 | 3 | translational termination |
| GO:0071243 | 0.040688 | 2 | cellular response to arsenic-containing substance |
| GO:0000022 | 0.04205 | 2 | mitotic spindle elongation |
| GO:0006810 | 0.046678 | 9 | transport |
| GO:0043001 | 0.048584 | 3 | Golgi to plasma membrane protein transport |
| GO:0051233 | 0.039574 | 2 | spindle midzone |
| GO:0031072 | 0.015503 | 4 | heat shock protein binding |
| GO:0015450 | 0.02355 | 3 | P-P-bond-hydrolysis-driven protein transmembrane transporter activity |
| GO:0070628 | 0.043322 | 2 | proteasome binding |

**TABLE S4** Unique significant enriched GO terms involved in wild type strain following salt stress challenge

| category | over_represented_pvalue | numDEInCat | term |
| --- | --- | --- | --- |
| GO:0006412 | 2.80E-16 | 50 | translation |
| GO:0000447 | 1.03E-08 | 17 | endonucleolytic cleavage in ITS1 to separate SSU-rRNA from 5.8S rRNA and LSU-rRNA from tricistronic rRNA transcript (SSU-rRNA, 5.8S rRNA, LSU-rRNA) |
| GO:0000463 | 2.73E-08 | 14 | maturation of LSU-rRNA from tricistronic rRNA transcript (SSU-rRNA, 5.8S rRNA, LSU-rRNA) |
| GO:0000472 | 5.30E-08 | 13 | endonucleolytic cleavage to generate mature 5'-end of SSU-rRNA from (SSU-rRNA, 5.8S rRNA, LSU-rRNA) |
| GO:0000480 | 5.58E-08 | 13 | endonucleolytic cleavage in 5'-ETS of tricistronic rRNA transcript (SSU-rRNA, 5.8S rRNA, LSU-rRNA) |
| GO:0006364 | 9.39E-07 | 19 | rRNA processing |
| GO:0042797 | 1.05E-06 | 9 | tRNA transcription from RNA polymerase III promoter |
| GO:0042254 | 8.61E-06 | 9 | ribosome biogenesis |
| GO:0042274 | 2.56E-05 | 7 | ribosomal small subunit biogenesis |
| GO:0000466 | 0.000138 | 7 | maturation of 5.8S rRNA from tricistronic rRNA transcript (SSU-rRNA, 5.8S rRNA, LSU-rRNA) |
| GO:0000027 | 0.000169 | 12 | ribosomal large subunit assembly |
| GO:0000462 | 0.000381 | 12 | maturation of SSU-rRNA from tricistronic rRNA transcript (SSU-rRNA, 5.8S rRNA, LSU-rRNA) |
| GO:0006360 | 0.001502 | 7 | transcription from RNA polymerase I promoter |
| GO:0031120 | 0.002271 | 4 | snRNA pseudouridine synthesis |
| GO:0000055 | 0.002389 | 5 | ribosomal large subunit export from nucleus |
| GO:0000056 | 0.00477 | 5 | ribosomal small subunit export from nucleus |
| GO:0042147 | 0.00778 | 5 | retrograde transport, endosome to Golgi |
| GO:0006534 | 0.009683 | 3 | cysteine metabolic process |
| GO:0019408 | 0.010511 | 3 | dolichol biosynthetic process |
| GO:0042790 | 0.012716 | 3 | transcription of nuclear large rRNA transcript from RNA polymerase I promoter |
| GO:0015691 | 0.014748 | 3 | cadmium ion transport |
| GO:0006661 | 0.014833 | 3 | phosphatidylinositol biosynthetic process |
| GO:0008272 | 0.015231 | 3 | sulfate transport |
| GO:0006874 | 0.015763 | 4 | cellular calcium ion homeostasis |
| GO:0036171 | 0.021686 | 5 | filamentous growth of a population of unicellular organisms in response to chemical stimulus |
| GO:0042493 | 0.023566 | 4 | response to drug |
| GO:0034727 | 0.02396 | 6 | piecemeal microautophagy of nucleus |
| GO:0000469 | 0.025254 | 2 | cleavage involved in rRNA processing |
| GO:0030150 | 0.025665 | 6 | protein import into mitochondrial matrix |
| GO:0009081 | 0.028994 | 5 | branched-chain amino acid metabolic process |
| GO:0018216 | 0.032182 | 2 | peptidyl-arginine methylation |
| GO:0006879 | 0.037725 | 7 | cellular iron ion homeostasis |
| GO:0071470 | 0.039747 | 7 | cellular response to osmotic stress |
| GO:0006913 | 0.04001 | 2 | nucleocytoplasmic transport |
| GO:0009113 | 0.041386 | 3 | purine nucleobase biosynthetic process |
| GO:0000379 | 0.043943 | 2 | tRNA-type intron splice site recognition and cleavage |
| GO:2000678 | 0.046679 | 2 | negative regulation of transcription regulatory region DNA binding |
| GO:0033566 | 0.046778 | 2 | gamma-tubulin complex localization |
| GO:0034244 | 0.047029 | 2 | negative regulation of transcription elongation from RNA polymerase II promoter |
| GO:0070676 | 0.04749 | 3 | intralumenal vesicle formation |
| GO:0006269 | 0.049172 | 2 | DNA replication, synthesis of RNA primer |
| GO:0071051 | 0.049999 | 2 | polyadenylation-dependent snoRNA 3'-end processing |
| GO:0005730 | 1.62E-27 | 93 | nucleolus |
| GO:0005762 | 3.71E-15 | 27 | mitochondrial large ribosomal subunit |
| GO:0005763 | 9.34E-10 | 15 | mitochondrial small ribosomal subunit |
| GO:0005736 | 7.18E-07 | 8 | DNA-directed RNA polymerase I complex |
| GO:0005666 | 1.22E-06 | 9 | DNA-directed RNA polymerase III complex |
| GO:0005761 | 7.27E-05 | 8 | mitochondrial ribosome |
| GO:0030688 | 0.000662 | 6 | preribosome, small subunit precursor |
| GO:0005783 | 0.002127 | 69 | endoplasmic reticulum |
| GO:0005852 | 0.007736 | 4 | eukaryotic translation initiation factor 3 complex |
| GO:0031429 | 0.008151 | 3 | box H/ACA snoRNP complex |
| GO:0034457 | 0.009173 | 3 | Mpp10 complex |
| GO:0031515 | 0.009806 | 3 | tRNA (m1A) methyltransferase complex |
| GO:0071540 | 0.009844 | 5 | eukaryotic translation initiation factor 3 complex, eIF3e |
| GO:0005656 | 0.010462 | 6 | nuclear pre-replicative complex |
| GO:0016282 | 0.01216 | 7 | eukaryotic 43S preinitiation complex |
| GO:0031966 | 0.017405 | 8 | mitochondrial membrane |
| GO:0000139 | 0.021663 | 7 | Golgi membrane |
| GO:0000329 | 0.023603 | 28 | fungal-type vacuole membrane |
| GO:0042645 | 0.026119 | 5 | mitochondrial nucleoid |
| GO:0001405 | 0.029211 | 3 | presequence translocase-associated import motor |
| GO:0071541 | 0.032014 | 4 | eukaryotic translation initiation factor 3 complex, eIF3m |
| GO:0015934 | 0.034344 | 2 | large ribosomal subunit |
| GO:0005665 | 0.038005 | 5 | DNA-directed RNA polymerase II, core complex |
| GO:0005654 | 0.040094 | 6 | nucleoplasm |
| GO:0005768 | 0.040443 | 11 | endosome |
| GO:0000214 | 0.043943 | 2 | tRNA-intron endonuclease complex |
| GO:0005720 | 0.047566 | 2 | nuclear heterochromatin |
| GO:0097344 | 0.047566 | 2 | Rix1 complex |
| GO:0001056 | 1.01E-07 | 9 | RNA polymerase III activity |
| GO:0001054 | 7.18E-07 | 8 | RNA polymerase I activity |
| GO:0030515 | 7.41E-06 | 8 | snoRNA binding |
| GO:0003729 | 4.96E-05 | 25 | mRNA binding |
| GO:0004004 | 0.000292 | 7 | ATP-dependent RNA helicase activity |
| GO:0008026 | 0.001114 | 17 | ATP-dependent helicase activity |
| GO:0001055 | 0.002268 | 5 | RNA polymerase II activity |
| GO:0019843 | 0.003162 | 5 | rRNA binding |
| GO:0003899 | 0.003817 | 9 | DNA-directed RNA polymerase activity |
| GO:0003968 | 0.008755 | 5 | RNA-directed RNA polymerase activity |
| GO:0008526 | 0.009409 | 3 | phosphatidylinositol transporter activity |
| GO:0016429 | 0.009806 | 3 | tRNA (adenine-N1-)-methyltransferase activity |
| GO:0003896 | 0.011507 | 3 | DNA primase activity |
| GO:0042134 | 0.011818 | 3 | rRNA primary transcript binding |
| GO:0017150 | 0.012348 | 3 | tRNA dihydrouridine synthase activity |
| GO:0015295 | 0.012564 | 3 | solute:proton symporter activity |
| GO:0043022 | 0.012619 | 5 | ribosome binding |
| GO:0015116 | 0.015231 | 3 | sulfate transmembrane transporter activity |
| GO:0015266 | 0.017229 | 5 | protein channel activity |
| GO:0008168 | 0.024904 | 10 | methyltransferase activity |
| GO:0003676 | 0.03302 | 72 | nucleic acid binding |
| GO:0016653 | 0.036564 | 2 | oxidoreductase activity, acting on NAD(P)H, heme protein as acceptor |
| GO:0005524 | 0.037083 | 87 | ATP binding |
| GO:0009055 | 0.037214 | 8 | electron carrier activity |
| GO:0000213 | 0.043943 | 2 | tRNA-intron endonuclease activity |
| GO:0008441 | 0.045118 | 2 | 3'(2'),5'-bisphosphate nucleotidase activity |


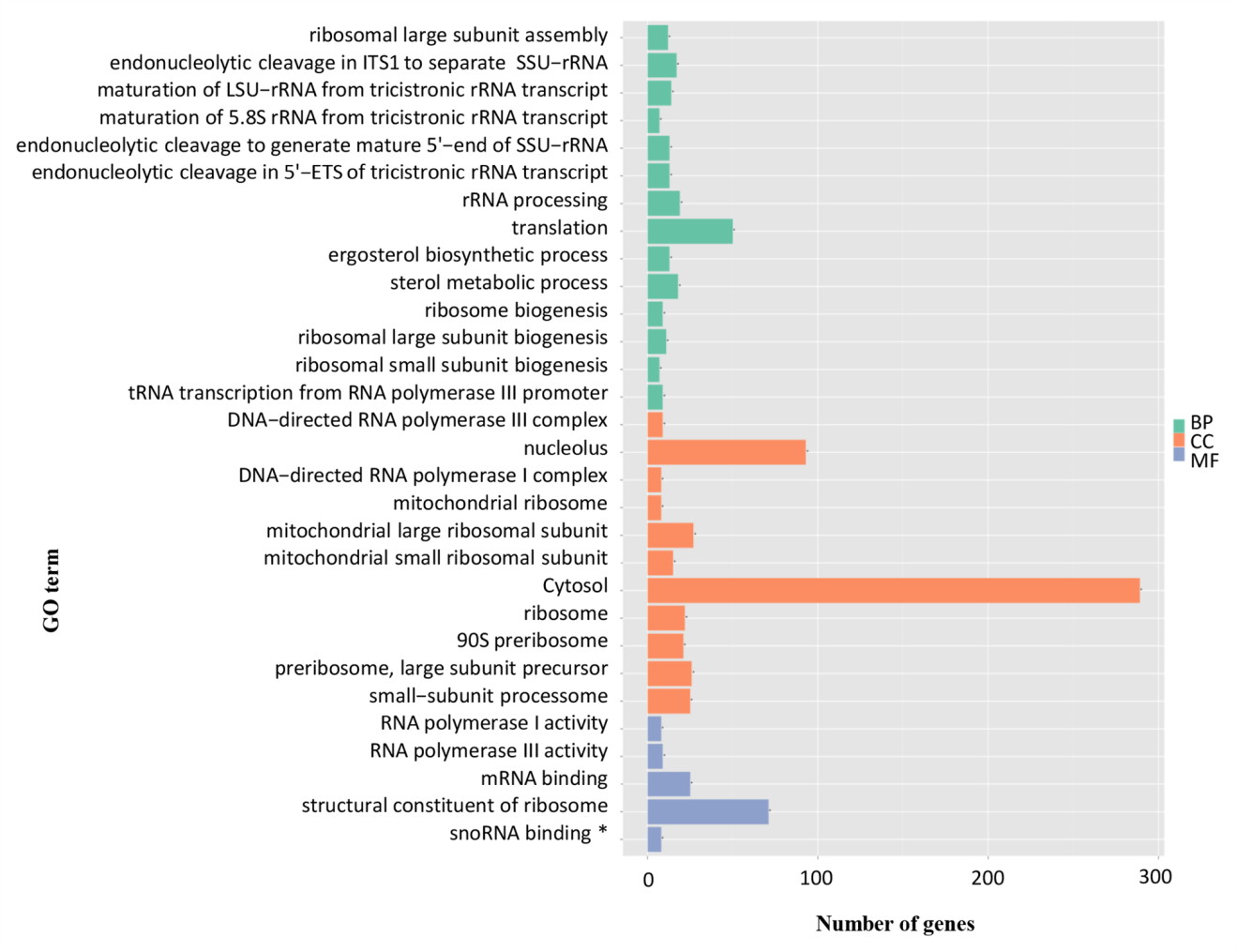


**Figure S1.** Most enriched GO terms among DEGs induced by salt stress in wild-type *A. nidulans*. For each enriched GO term with a Bonferroni P-value < 0.01, the ontology to which the GO term belongs is shown (BP = Biological Process; CC = Cellular Component; MF = Molecular Function).


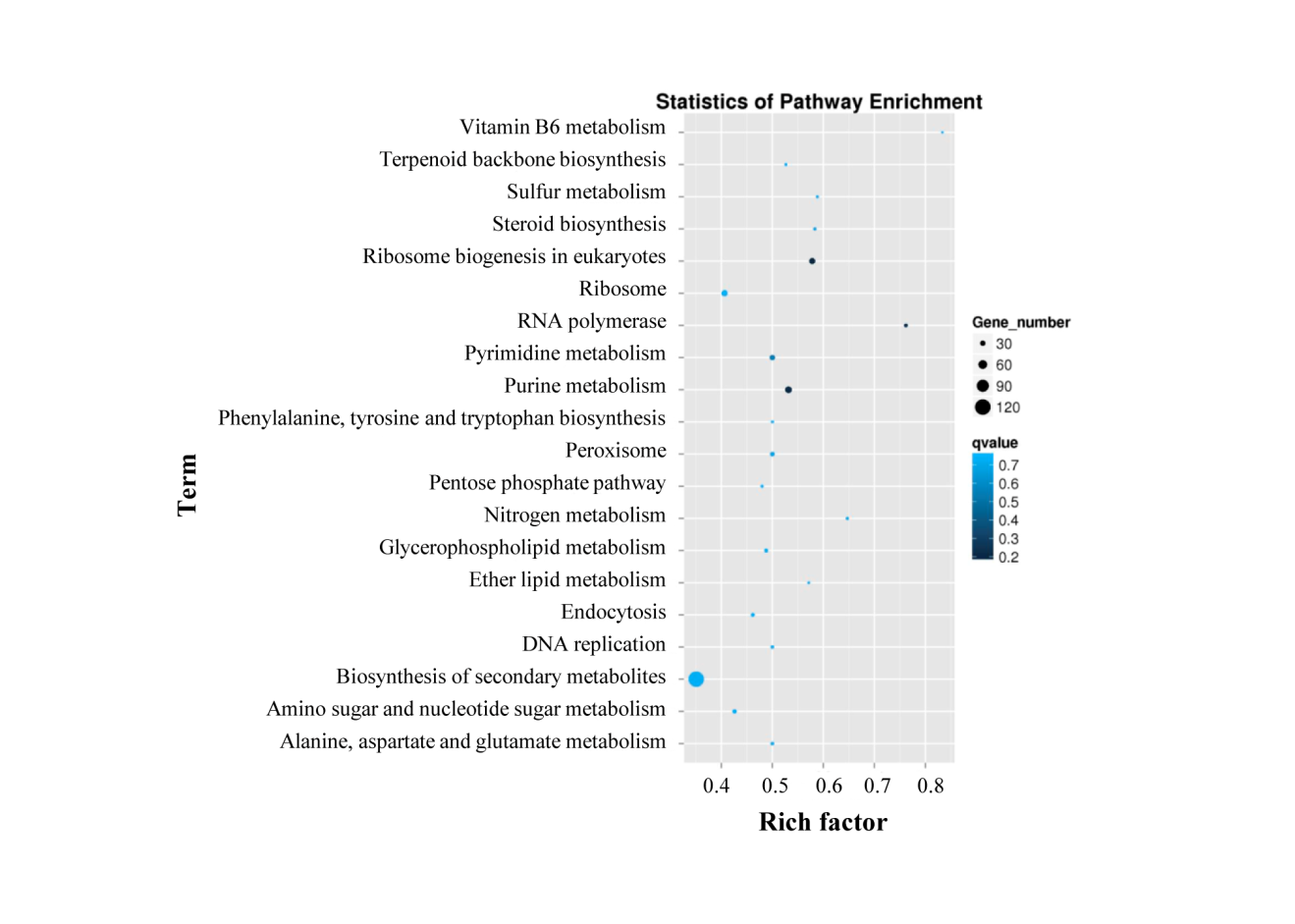


**Figure S2.** KEGG pathway enrichment of DEGs induced by salt stress in wild-type *A. nidulans*. The x-axis indicates the enrichment factor of each pathway, and the y-axis indicates each pathway.
